# PROMOTING CO-WORKER RELATIONSHIPS IN LONG-TERM CARE FACILITIES: A SYSTEMATIC REVIEW OF THE LITERATURE

**DOI:** 10.1093/geroni/igad104.2854

**Published:** 2023-12-21

**Authors:** Kirsty Haunch

**Affiliations:** University of Leeds, Leeds, England, United Kingdom

## Abstract

**Introduction:**

Co-worker relationships in long-term care settings are important. The work is physically and emotionally demanding. A largely unregistered workforce with variable preparation and training are expected to provide complex care to the most vulnerable members of society, with little external support. Often staff only have each other to rely on. Current evidence lacks insight into how co-worker relationships are established and what influences them. The aim of the review was to synthesise what is known about co-worker relationships more widely and its applicability to long-term care.

**Methods:**

A systematic review identified, evaluated, and synthesised published evidence associated with co-worker relationships in health and social care settings. PRISMA guidelines were followed. The COM-B model was used to identify key behaviours and associated triggers of co-worker relationships.

**Results:**

37 papers were included. The majority of studies were conducted in the US (n=23), used descriptive study designs (n=30) and were in a hospital setting (n=29). Interdependent organisational and individual factors suggest co-worker relationships occur when staff are ‘able’ (have the social skills), are ‘willing’ (have the right values) and are ‘supported’ (effective leadership).

**Discussion:**

To date the focus has been on the ‘willingness’ of people and the ‘support’ they receive. Paying attention, the ‘ability’ (the social skills), which can be taught, is of equal, if not more importance. Social skills enable staff to navigate difficult and complex situations that arise, which preserves, and in some cases strengthens relationships. Social skills are particularly important when leadership is poor.

